# Supplementary material for: Analysis of chi angle distributions in free amino acids via multiplet fitting of proton scalar couplings
Source: Magn Reson (Gott). 2024 Aug 19;5(2):103–20. doi: 10.5194/mr-5-103-2024 (PMC11570886; doi:10.5194/mr-5-103-2024)
Supplement: The supplement related to this article is available online at: https://doi.org/10.5194/mr-5-103-2024-supplement. [file mr-5-103-2024-supplement.zip › Syed2024/data/fit1d_fitnmr_output.tar.gz!/fit1d/thr/500/bmse000049_simulation_1_nmredata/1H/resonances_1d.pdf]

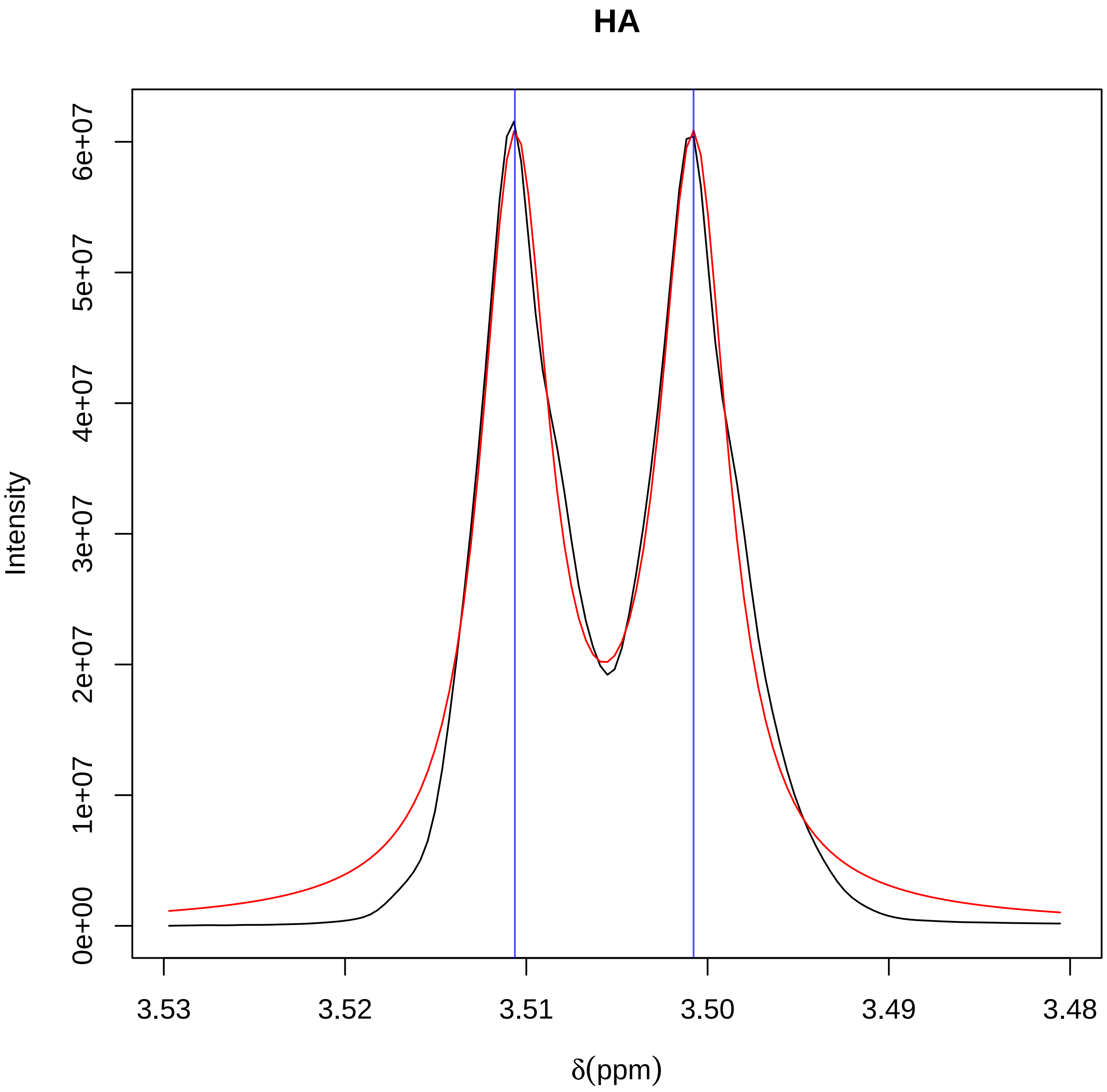

HA
Intensity
0e+00
1e+07
2e+07
3e+07
4e+07
5e+07
6e+07
3.53
3.52
3.51
3.50
3.49
3.48
δ(ppm)

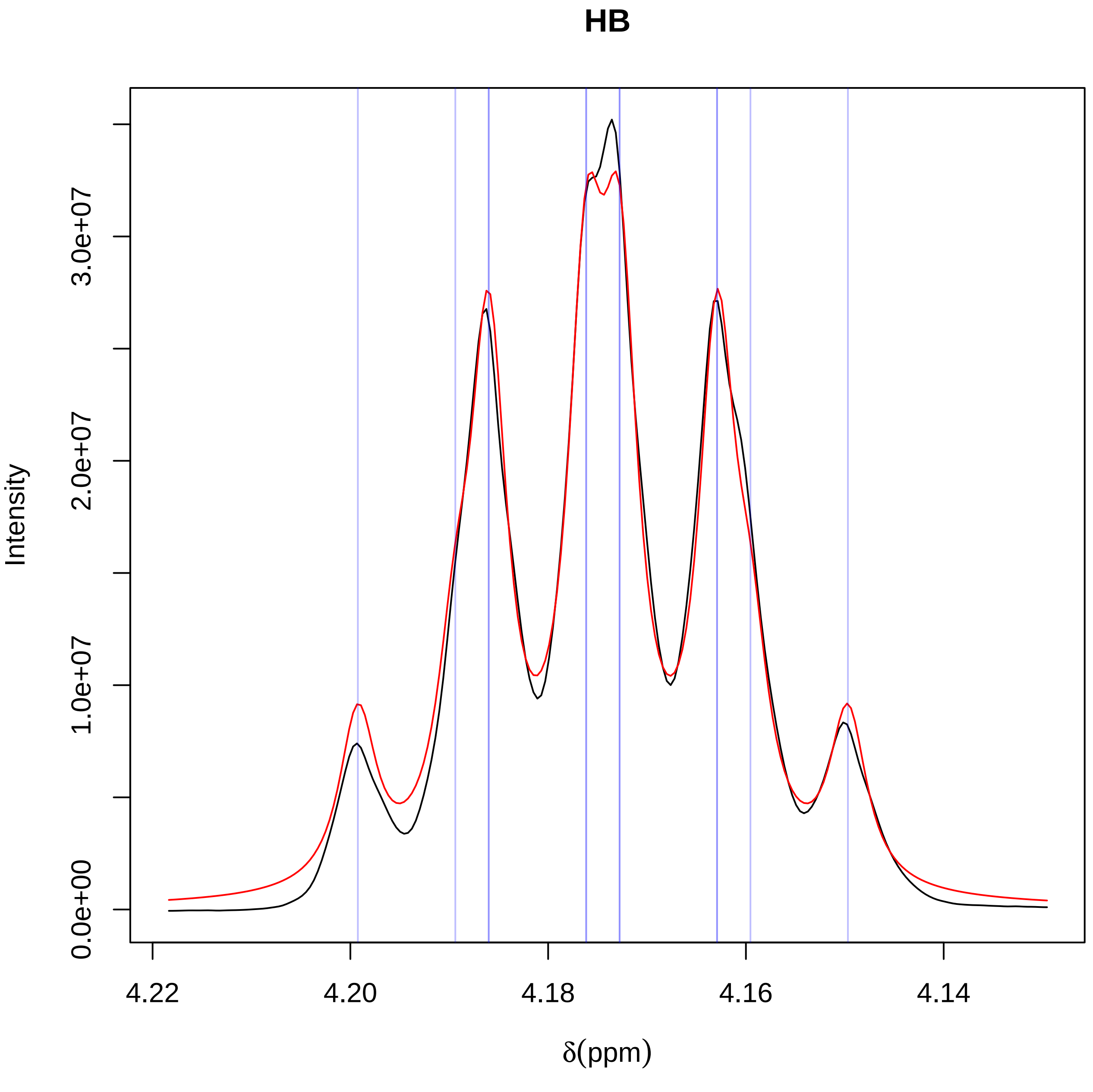
HB
Intensity
0.0e+00
1.0e+07
2.0e+07
3.0e+07
4.22
4.20
4.18
4.16
4.14
δ(ppm)

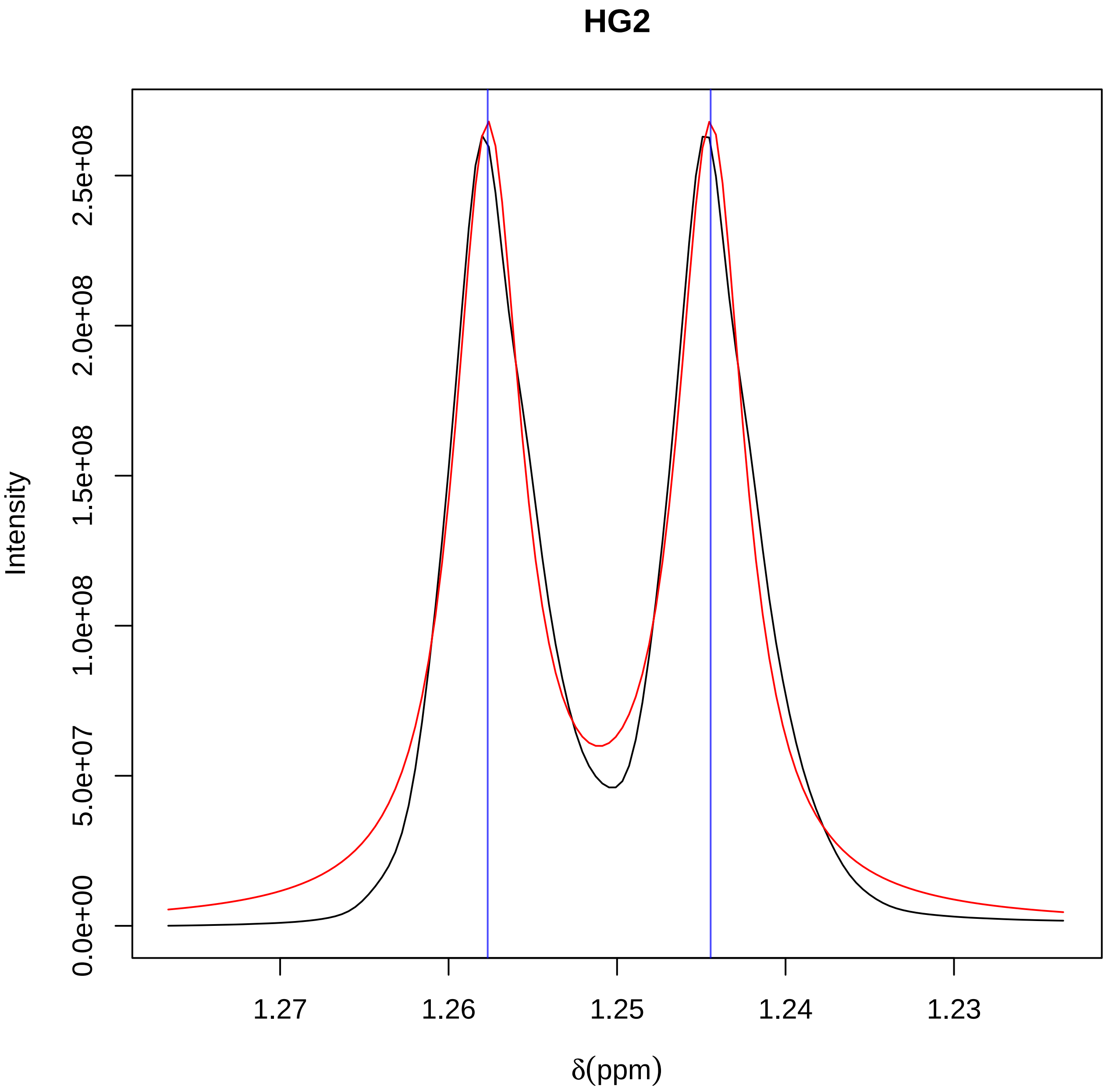

HG2
Intensity
0.0e+00
5.0e+07
1.0e+08
1.5e+08
2.0e+08
2.5e+08
1.27
1.26
1.25
1.24
1.23
δ(ppm)
